# The Pioneers of America's United Effort

**Published:** 1918-04-06

**Authors:** 


### THE AMERICAN RED CROSS.

### The Pioneers of America's United Effort.

Mr. Henry Pomeroy Davison, Chairman of the War Council of the American Red Cross, and in private life a member of the firm of J. P. Morgan and Co., has given a remarkable account to a representative of the *Times* of the extent of the help which America is giving to the Allies through the gigantic development of the American National Red Cross. The figures are American in size and American in number, but there is more than mere vulgar amazement to be read in them. For they represent the mammoth resources of the Republic, the huge range of nationalities which compose the American nation, and the restless energy of the immense human swarm which is now seething with activity in every department of work on behalf of the Allied cause. There is a dignity and even a restraint in Mr. Davison's explanation of his figures which will do more than the mere size of his organisation to bring home America's part in the war to us in Europe. He says: "There is no question of charity. It is a question of recognising the situation and doing whatever may be done by the people who are to-day the richest in the world—I do not say that offensively; it is an embarrassment rather than anything else as I see it." Opportunity can make men look foolish by mere comparison of her gifts and with their capacity. That has been the difficulty of America from the first. Her occasions were so immense that she could not live up to them; her wealth so great that it made a fool of her people. Since in point of view of wealth she began where Europe had left off, and, unlike Europe, was crippled by the absence of any tradition which might enable her, as it had taught the rich here, to carry their rank and wealth lightly in view of the obligations which traditionally attached to both in Europe, America, in short, has been largely a place where there was little for men to do except to make money, and, having made it, few public services for them to enter to shoulder the duties which always await new recruits to the aristocracy in Europe. Thus an American, having made money, found no such reward as was open to his public spirit here. He looked round in vain for something to do, and having never had or understood the habit of leisure, which he always confused with idleness, he felt a fool, and, in despair, went back to his office or his shop to prevent himself from perishing of uselessness. This, or something like it, is behind Mr. Davison's admission that wealth has been an embarrassment to America. His story of the growth of the Red Cross organisation is an example of America's attempt to escape from this embarrassment. So understood, the figures which he gives are something more interesting than an endless row of ciphers and integers.

Last June twenty millions were raised in one week as a War Fund for the Red Cross, and in the past six months ten millions have been spent in various forms of work for the French people alone. These sums refer to pounds, not to dollars. The subscribing members of the American Red Cross now number 23,000,000, or more than one hundred times as many as it possessed ten months ago.

In addition to our knowledge of its work for the wounded soldiers in France, some idea should be given of the range of organisations now in being for the benefit of the French, including the civilian population. The problems in France include those of the homeless families and the orphan children, of the spread of tuberculosis, which has been encouraged by the war, and of the devastated areas. In ways too numerous to be described otherwise than by a catalogue of activities, the American Red Cross Society is tackling all these problems on the spot. Fifty thousand homeless and orphan children in France are being cared for; the homes of soldiers in devastated areas to which they are returning when on leave are being repaired, rebuilt, and refurnished for them. Their very families are being reinstated. Tuberculosis among the civilian population is being attacked by means of travelling dispensaries and a supply of trained nurses for the sick. The population question, too, is not forgotten. Expectant and nursing mothers and the children of the latter are being regularly fed. It is, indeed, hardly too much to say that the American Red Cross on its non-military side is almost in itself a ministry of health with multifarious organisations. When we turn to its military activities we have only to mention that the American Society is maintaining over fifteen hospitals in France and five in England, that it is contributing supplies and money to 4,000 military hospitals in France and here, and that by next June the *personnel* of the mission to France, which numbered eighteen a year ago, will reach 5,000. Three days after the Italian disaster in the autumn the American organisation was already in charge of 600,000 refugees, and its flag was to be seen in almost every Italian city. Where the flag flew, there was the centre of the local organisation.

Mr. Davison claims, no doubt with justice, that the work done by the American Red Cross in the care of the French soldier while on leave from the trenches has raised his *morale*, as such proofs of help were bound to do. The French soldier has been taught by personal experience that America is thereby in the war, and that where the Red Cross organisation has led the way the American Army will soon follow. To American resource the French soldier may find that he owes the repair of his home, the reunion of his scattered family, and the supplies on which that family depends. Fraternity, a word better understood in France than in England, will take a new lease of affection in the soldier's mind. He is experiencing the fruits of the Alliance.

If, as we have ventured, Englishmen may study these figures as much for the revelation which they provide of the meaning of the war to America, as for the sake of some idea of the organisation sug-

**THE AMERICAN RED CROSS**—(*continued*).

gested by them, a better mutual understanding will result. Even now most Englishmen do not realise the immensity of the conversion which the entry of the States into the war implied. They think that the English-speaking world was bound to join together in so great an issue, and perhaps that America was slow in coming in. Such folk have no conception of the tradition and habit of peace which animated America. War to her, in a very limited and remote experience, was of its nature "civil war"—that is to say, the worst and most appalling of human evils. We can therefore understand what Mr. Davison means when he declares that the American Red Cross is not merely a recognised organisation performing a departmental work in the war. We can understand that it is no rhetorical phrase to describe it, as he does himself, as "the mobilised spirit and heart of the American people." America has learnt the lesson which it took England herself a long time to learn—that the blessings of peace are not merely to be enjoyed and preserved by peaceful activities, but are themselves to be fought for in the last resort. The American Red Cross, therefore, is the first army which the United States have been able to place in the field. Its work is the proof of America's determination; its extent is the measure of her resources; its beneficent aims the symbol of the temper in which she enters the great struggle. And who can doubt that, in the long run, her allegiance more than makes up for the amorphous millions of Russia, and is a fitter Ally for European democracy, than the autocratic government of the Czar?

